# A probabilistic sampling method (PSM) for estimating geographic distance to health services when only the region of residence is known

**DOI:** 10.1186/1476-072X-10-4

**Published:** 2011-01-10

**Authors:** Kirsten MM Beyer, Audrey F Saftlas, Anne B Wallis, Corinne Peek-Asa, Gerard Rushton

**Affiliations:** 1 Institute for Health and Society, Medical College of Wisconsin, 8701 Watertown Plank Road, Milwaukee, WI, 53226, USA; 2 Department of Epidemiology, University of Iowa, C-21M General Hospital, Iowa City, IA 52242, USA; 3 Department of Occupational and Environmental Health, College of Public Health, University of Iowa, 100 Oakdale Campus #114 IREH, Iowa City, IA 52242, USA; 4 Department of Geography, University of Iowa, 316 Jessup Hall, Iowa City, IA 52242, USA

## Abstract

**Background:**

The need to estimate the distance from an individual to a service provider is common in public health research. However, estimated distances are often imprecise and, we suspect, biased due to a lack of specific residential location data. In many cases, to protect subject confidentiality, data sets contain only a ZIP Code or a county.

**Results:**

This paper describes an algorithm, known as "the probabilistic sampling method" (PSM), which was used to create a distribution of estimated distances to a health facility for a person whose region of residence was known, but for which demographic details and centroids were known for smaller areas within the region. From this distribution, the median distance is the most likely distance to the facility. The algorithm, using Monte Carlo sampling methods, drew a probabilistic sample of all the smaller areas (Census blocks) within each participant's reported region (ZIP Code), weighting these areas by the number of residents in the same age group as the participant. To test the PSM, we used data from a large cross-sectional study that screened women at a clinic for intimate partner violence (IPV). We had data on each woman's age and ZIP Code, but no precise residential address. We used the PSM to select a sample of census blocks, then calculated network distances from each census block's centroid to the closest IPV facility, resulting in a distribution of distances from these locations to the geocoded locations of known IPV services. We selected the median distance as the most likely distance traveled and computed confidence intervals that describe the shortest and longest distance within which any given percent of the distance estimates lie. We compared our results to those obtained using two other geocoding approaches. We show that one method overestimated the most likely distance and the other underestimated it. Neither of the alternative methods produced confidence intervals for the distance estimates. The algorithm was implemented in R code.

**Conclusions:**

The PSM has a number of benefits over traditional geocoding approaches. This methodology improves the precision of estimates of geographic access to services when complete residential address information is unavailable and, by computing the expected distribution of possible distances for any respondent and associated distance confidence limits, sensitivity analyses on distance access measures are possible. Faulty or imprecise distance measures may compromise decisions about service location and misdirect scarce resources.

## Background

Epidemiologists and public health policy researchers share an interest in studying geographic access to health services, especially for high-risk populations and underserved communities. Computing geographic access, however, is complicated by the fact that many data sets contain geographic information such as the census tract, ZIP Code or county of residence instead of precise residential address information. Because of the geographic and demographic heterogeneity of these areas, distance calculations may be imprecise with no information about the most likely range of distances within which the true distance will lie (confidence limits). Ascertaining the best estimate of geographical access to services afforded by available data is critical to efforts aimed at improving health access. Imprecise estimates, however, can compromise the efficacy of policy and programmatic decisions and may misdirect scarce resources.

There is a sizeable literature that seeks to improve estimation of geographic access to health care services [1] and to assess the potential errors in the methods currently in use [2]. Methods for calculating distances between an individual and a facility include Euclidean (straight line) and network distance calculations. Network distance calculations take into account street networks and may incorporate other data, such as speed limits or the time it takes to make a turn. At present, Euclidean distance is the most widely used method; however, it is flawed because people travel by real transportation networks, not "as the crow flies" [1]. Increasingly, web-based distance estimating algorithms are available to provide more accurate distance estimates, but it is common for a user to know only the area within which a person lives rather than their exact location. This problem is generally solved by assigning a geocode representative of the area in question to the individual, which raises the question of determining the most appropriate geocode for this purpose.

Determining the best geocode to use in calculating distance is a common problem [3,4]. It has been common practice to use the centroid - or geometric center - of a geographic area as a point location representing the residence of an individual known to reside within that area, when no further information is available. Often, the ZIP Code is the best available geocode in a dataset. However, use of a ZIP Code area centroid as a representation of a person's location within that ZIP Code can be problematic for several reasons.

First, it is difficult to locate the centroid of a ZIP Code, as true boundaries of ZIP Codes are generally not known. US ZIP Codes were defined as routes for the delivery of mail, not as distinct geographic areas, and in some cases they do not even form spatially contiguous areas. Since 2000, ZIP Code centroids have been calculated using a new US Census Bureau geographical unit that establishes ZIP Code boundaries: the ZIP Code Tabulation Areas (ZCTAs™). The ZCTA was developed to estimate the geographical area represented by a ZIP Code. ZCTAs are created from Census blocks, where the ZIP Code of the majority of residents in a Census block determines that block's membership within a ZCTA. The merits and detriments of the ZIP Code as a geocode have been discussed at length previously [5-10].

A second weakness is that the ZCTA centroid represents the center of land area within a ZIP Code, which may not be a population center. The resultant centroid location may be non-representative of population density (e.g., very few people may live there) or it may be the location of a lake or other non-residential area. Previous work has sought to address this problem by placing the geocode not at the centroid of the ZCTA, but at the centroid of the largest incorporated area within the ZCTA - the ZIP Code population center - arguing that this better represents the location of an individual's true residence [6]. It is well known that if the largest population center within a ZIP Code is larger than one-half of the population of the ZIP Code, it is the median population center of the ZIP Code area no matter where it is located within the ZIP Code area.

We conclude that there are serious limitations and some undesirable consequences of using current methods for estimating distances from people whose locations are known only by the areas within which they live. However, we have access to information about the population distribution within a ZIP Code that, to some degree, can be used to address these limitations.

In this paper, we present a method which utilizes knowledge of the distribution of people with their area of residence for estimating geographic access to a specified health service for a defined user-group, thus making it possible to improve efforts to equitably distribute needed services. Specifically, we (1) describe a probabilistic sampling approach to approximate an individual's geographic location, when only a region is known, and estimate the most likely distance the person would have to travel to access services, (2) compare the results of our method to those obtained by two other distance-estimating approaches, and (3) show that the two methods over-estimate and underestimate respectively, the distance of the person to the health facility. We apply these methods to a sample of women screened for intimate partner violence (IPV) in a reproductive health clinic to identify the distance from each of the women to the closest domestic violence intervention facilities, with a focus on rural women. Since this was an anonymous screening, individual addresses were not collected, although participants did report their ZIP Code of residence.

## Results

### Algorithm

To estimate the distance a person (e.g. a survey respondent) would have to travel to reach their nearest facility when only their region of residence (e.g. ZIP Code) is known, we first identified the smaller areas (e.g. Census Blocks) nested within that region, for which detailed demographic information and centroids were available. Our algorithm selected 30 sampled locations (including up to 30 different smaller areas) within the larger region using probability proportional to size sampling with replacement. This sampling strategy selects one smaller area from within the larger region, with each area's probability of selection weighted by the number of women of the respondent's age living in that area; the area selected is then "replaced" back into the set of possible areas for selection, and the sampling procedure is repeated until a sample size of 30 is obtained. This means that an area's probability of being selected during the first iteration is the same as its probability of selection in each subsequent iteration, making each selection independent of the others. An area could be selected more than once or not at all. We next identified the centroid (geometric center) of each smaller area sampled, and calculated distance along the road network between each of the 30 centroids of the smaller areas and the nearest facility. This process resulted in a population-weighted distribution of distances that represent the distances each respondent might have to travel. We then identified the median distance, bounded by 93% confidence intervals, as the most likely distance the respondent would have to travel to access services, as this provides a measure of central tendency that is resistant to outliers in addition to a measure of certainty regarding the estimate. The use of the median - as opposed to the mean - is common practice in location science because the median of a spatial distribution is the point that minimizes the sum of the distances of people to itself, whereas the mean minimizes the sum of the squared distances of people to itself. The 93% confidence interval was employed here for the sake of convenience, due to the fact that we sampled a total of 30 locations; the interval presented is bounded by the second (shorter) and 29^th ^(longer) distances in the distribution of 30 distances calculated for each woman. Alternative definitions and thresholds could be employed for the presentation of measures of "confidence" or "range" associated with our median distance measure. Note that all estimated distances were calculated to the closest health facility from each smaller area within the region, and therefore estimated distances from any region were not necessarily to the same health facility. In order to maintain the probabilistic nature of the method, we re-sampled for each respondent even if more than one respondent resided in the same region. This re-sampling approach was selected over the alternative deterministic approach, which could have established precisely the proportion of persons of the same demographic characteristics as the person of interest at different distances from the smaller areas, because we wanted to estimate the likelihood of a given person on a given occasion being at a given distance from their nearest health facility. The performance of a set of facilities for serving a given population can be better evaluated with reference to possible distances than by reference to average distances. Possible extreme distances matter in accessing the viability of particular facility location decisions.

### Testing

We tested our method using a study sample of participants in an anonymous clinic-based survey designed to estimate the prevalence of IPV among women seeking abortion services at a large clinic in Iowa [11], considering the survey respondent's ZIP Code as her "region" and the Census Blocks within that ZIP Code as the "smaller areas." It is known that women seeking abortions are at increased risk of IPV [12-14], and that geographic isolation and access to domestic violence services can pose difficulties in rural areas [15-17]; thus, we were interested to know how far these high risk women, located throughout a rural state, would have to travel to access domestic violence intervention services, in order to determine whether distance to services may constitute a potential barrier to addressing the problem of IPV among this population. We sought to provide the best estimate of geographic access to services given our limited knowledge of only a survey respondent's age and ZIP Code - a common problem in public health research. An analysis exploring geographic disparity in access to domestic violence services using distances generated by the method we describe was complete and nearing submission for peer review at the time of this writing.

Data were collected between November 2006 and July 2008. The study sample of 1501 respondents included data on ZIP Code of residence and age. Of the original 1501 respondent records, 22 were deleted due to missing data (age, ZIP Code, or both). In addition, sixty-one records contained ZIP Codes not included in Iowa's 949 ZCTAs; we reassigned 52 of these records to ZCTAs by identifying their locations through the USPS ZIP Code Lookup tool; 9 records were deleted because no ZCTA could be determined using the lookup tool or because the ZIP Code was for an out-of-state location. We discovered 3 cases in which ZCTA population data contained no women of the respondents' age; therefore, it was impossible to perform the sampling procedure, and the records were deleted. The final dataset for which the sampling procedure was performed included 1467 records.

We used respondents' age and ZIP Code in concert with US Census 2000 population data to estimate location of residence. Geographic areas used were ZIP Code/ZCTA to represent the known residence "region" and the Census block to represent the "smaller areas" within the region. In 2000, there were 949 ZCTAs in the state of Iowa, each created based on Census blocks and intended to estimate the area covered by a particular ZIP Code. Not all valid ZIP Codes are represented by a ZCTA; some may fall within a ZCTA associated with a different ZIP Code. Geographic boundaries of Iowa Census blocks were obtained from the ESRI data center http://arcdata.esri.com/data/tiger2000/tiger_download.cfm and ZCTA boundaries were obtained from the US Census Bureau website http://www.census.gov/geo/www/cob/z52000.html. Census data on age and gender were obtained for Iowa Census blocks (Census 2000 table P12). http://factfinder.census.gov/servlet/DownloadDatasetServlet?_lang=en. Study participants were females 18-44 years of age.

We performed our sampling procedure using ZCTAs as "regions" and Census Blocks contained within them as "smaller areas", as described above. Code was written and implemented using R software using the ppswr() function available in the pps package [18].

**Figure 1:**
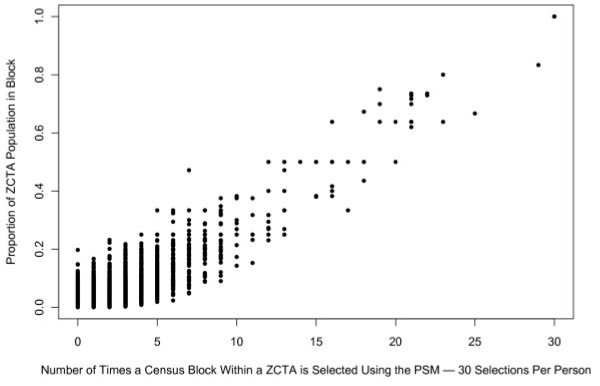
**Multiple selection increases with proportion of ZCTA population of women of respondent's age in the census block**.

To validate our sampling method, we plotted all participants to show the proportion of the ZCTA population in each Census block with the number of times the block was selected using the PSM. Figure 1 shows that the blocks were sampled proportionate to the ZCTA's age-specific population within a block (i.e., ZCTAs with few women of reproductive age had fewer sampled blocks than those with more women of reproductive age). We also inspected the samples visually to confirm that the sampling was probabilistic and not deterministic (i.e., that not all blocks with a proportion of .33 population are sampled exactly 10 times), and confirmed that there were 30 blocks sampled for each respondent.

### Implementation

We geocoded 28 IPV resource center locations provided by Children & Families of Iowa Family Violence Center, who maintain and update annually a compendium of state IPV resources. Locations were identified by a street address or a Post Office (PO) box number with city and ZIP Code. We geocoded 14 locations with address information using ESRI ArcMap. Of these 14, 10 were geocoded with 100% accuracy, and 4 with 81% accuracy. Locations were geocoded using the street network of Iowa obtained from the Iowa Natural Resources GIS Library http://www.igsb.uiowa.edu/nrgislibx/, which is based on the US Census TIGER/Line files for 2000. The remaining 14 records had only PO boxes and ZIP Codes; for these, the resource center was placed at the centroid of the largest incorporated area in the ZIP Code--a method described in more detail elsewhere [6]. We calculated distances along the road network between the sampled block centroids and the service center locations, using ESRI ArcMap™ software (including the Network Analyst extension). This resulted in a distribution of 30 distance estimates for each individual. We then selected the median distance in this distribution as the most likely distance an individual would have to travel to access IPV services and calculated the 93% confidence intervals to identify the likely range of distances.

It is important to consider how this distance estimation method compares to other common approaches to estimating distances when only the ZIP Code of residence is known. A common practice is to use the centroid of an individual's ZCTA as their geocoded residence. Another approach is to select the centroid of the largest incorporated area in an individual's ZIP Code/ZCTA, a method described previously [6]. We calculated two new sets of network distances to IPV service locations, using these two alternative methods, and used linear regression analyses to predict the median distance obtained using our PSM with the ZCTA centroid and ZIP Code center distances, respectively

**Figure 2:**
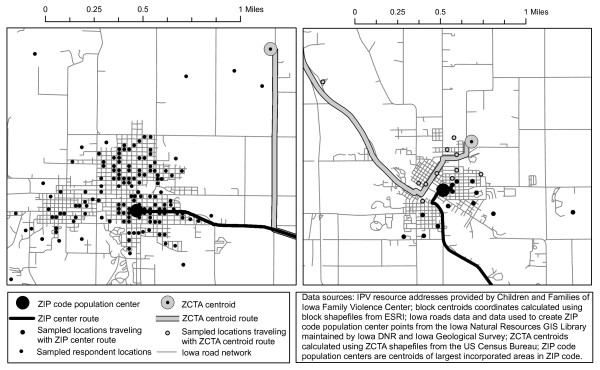
**The effects of residential geocode on distance estimation and determination of closest facility**. PANEL 1 shows that in some cases, the ZIP Code center is much closer to representing the geography of the sampled locations than the ZCTA centroid, although the effect on distance estimation may be negligible. PANEL 2 illustrates that respondents may be comparably close to multiple service centers. Use of ZCTA centroid or ZIP Code center geocode alone may result in different determinations of which facility is closest. A probabilistic sampling approach includes all facilities to the degree that they are relevant.

Figure 2 illustrates how these three distance estimation approaches differ. The panel on the left shows the difference between the ZCTA centroid and the ZIP Code population center methods, where the ZIP Code center point more closely corresponds with the locations of the sampled blocks. In Panel 2, depending on the geocode (ZCTA centroid vs. ZIP Code center), a different service center is identified as being the closest; when our PSM is used, both facilities are represented in the calculation, with the number of times they are included reflecting their likelihood of the IPV facility being closest to the subject's residence.

Our discussion of the results of this comparison of distance estimates focuses on three measures of performance: (1) summary measures of the statistical distributions of the distance estimates, (2) measures of the confidence limits for the PSM distances, and (3) measures of the differences between the estimates from the three methods.

#### 1. Summary Measures of the Estimated Distances

Results of the three distance calculations are shown in Table 1. For the purposes of comparison, we include both the mean and median of distances calculated using our PSM distance estimation approach, along with ZCTA centroids and ZIP Code population centers as geocodes. It is important to note that the minimum distance value calculated using the ZIP Code population center point as a geocode for the woman's residence is 0 because some IPV service locations were also geocoded to the ZIP Code population center when street address information was unavailable. Likewise, some distances calculated using ZCTA centroid points are essentially calculations of the distance between the ZCTA centroid and the ZIP Code population center, if IPV service centers were geocoded to the ZIP Code center points and women's residences were geocoded to ZCTA centroids.

**Table 1:** Measures of central tendency for estimated distances (in miles) between respondent locations and IPV service centers using three methods

	PSM (median)	PSM (mean)	ZCTA centroid as geocode†	ZIP Code population center as geocode
Range	0.39 - 66.4	0.53 - 65.6	0.09 - 67.2	0.00* - 66.6

Median	5.2	5.4	5.4	6.0

Mean (SD)	10.1 (11.2)	10.2 (11.2)	10.5 (11.1)	9.9 (11.2)

1^st ^Quartile	2.9	3.1	3.6	3.4

3^rd ^Quartile	12.2	12.3	12.1	11.7

The table compares distance estimates obtained using the Probabilistic Sampling Method (PSM), ZCTA centroid, and ZIP Code population center geocoding approaches (n = 1467). * The value of 0 reflects the distance (e.g., 0 miles) between the ZIP Code center point and itself when it is used as a geocode for both the subject's residence and the location of the IPV service center. † Some distances calculated using the ZCTA centroid as the geocode for the subject's residence reflect the distance between the ZCTA centroid and the ZIP Code center point, when IPV service centers were geocoded to ZIP Code centers.

In our sample, the distribution is skewed toward shorter distances, with a minimum distance traveled of 0.39 miles and a maximum of 66.4 miles. The median distance to the closest service facility for all women was 5.2 miles using the PSM. The maximum number of facilities used for estimating distance for a single respondent was 4, which again was not a frequent occurrence, as the mean number of facilities used to calculate distances was 1.66, and the median was 1.

**Figure 3:**
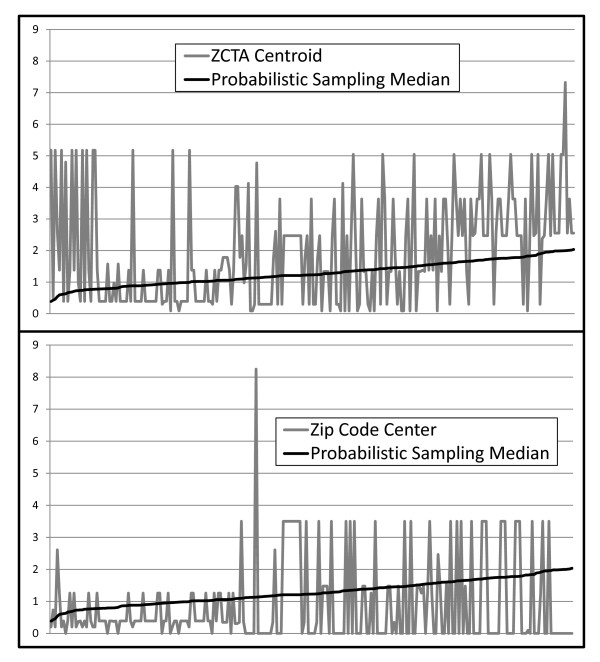
**Comparison of the PSM distances with two commonly used alternative methods for estimating distances between residence and service location**.

Estimated distances vary depending on the geocode used, and differences among methods are most apparent at shorter distances, which make up the vast majority of distances calculated. Figure 3 illustrates the variability of distances calculated using the ZCTA and Zip Code population center methods for the 150 women with the shortest distances to their nearest IPV center as calculated by the PSM median distance method. As both graphs order the observations by the PSM median distance calculated for each individual and use the same value scale, the graphs can be directly compared. Both the ZCTA centroid and ZIP Code center distances vary significantly from the median of sampled distances. This variability of the distance estimates in the short distance range shows that ZCTA centroid and ZIP Code center distance calculations do not take into account the known distribution of the distance estimates around any single case. In general, the ZIP Code center geocode appears to exhibit less variability, which can be attributed to the fact that it is deliberately placed in a populated area. However, in some cases it may be more problematic than the ZCTA centroid, for example, in situations where there are two significant populated places within a ZIP Code, and a woman is geocoded to the larger of these places when in fact she actually resides in the smaller one; the ZCTA centroid may be a better approximation of her actual location in this situation.

#### 2. Measures of the confidence limits for the PSM distances

**Figure 4:**
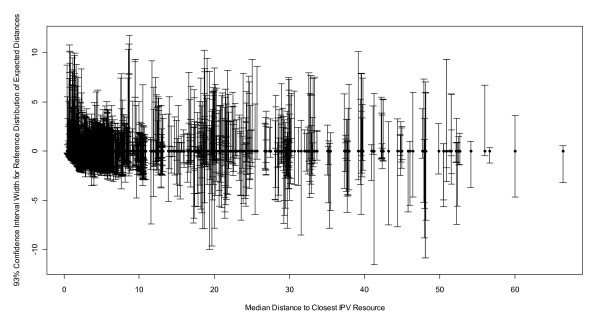
**Width of the 93% confidence interval increases with median distance to closest IPV resource (PSM method)**.

Figure 4 shows the 93% confidence intervals for each person using PSM. These figures show tighter confidence intervals (greater precision) for shorter distances (e.g., more urban service areas) and wider confidence intervals for greater distances (e.g., more rural locations). Neither the ZCTA centroid nor ZIP Code center calculations provide confidence estimates.

#### 3. Measures of the differences between the estimates from the three methods

**Figure 5:**
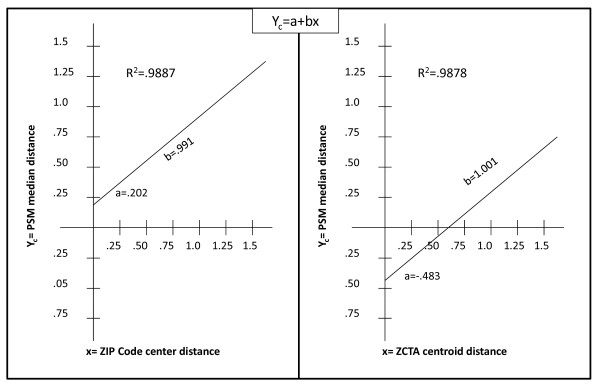
**Predicting the PSM median distance with ZCTA centroid and ZIP Code center distances**.

We measured the consistency of the distance estimates by the three methods by regressing each median PSM distance estimate against the distance estimate of the ZCTA centroid and Zip Code center respectively. Figure 5 shows the results of these regressions. In both cases the statistical fit (R^2^) is very high and the linear coefficient very close to 1.0. However, in both cases it is evident that the estimated distances are biased in relation to the distances estimated by the PSM median distance method. In general, the distances computed using the ZIP Code population centers underestimate the distances and the distances using the ZCTA centroids overestimate them.

These results can be generalized to other efforts to measure spatial access measures to health facilities. The theoretical literature in geographic information science concludes that the size of the over- and under-estimation is, in general, a function of the ratio of the number of areal units to the number of health facilities [19]. As the number of ZIP Codes per IPV facility increases, the number of participants allocated to more than one IPV facility will decrease, within the boundaries of their ZIP Code using the PSM.

In the Iowa study region where we used the PSM, there were 218 ZIP Codes and 28 IPV facilities--a ratio of approximately 8:1. For either of the two comparison methods, as this ratio decreased, the overestimation of distances increased. In this implementation, the ratio of approximately 8:1 leaves one method to underestimate distances and the other method to overestimate them. Sensitivity of results to the value of this ratio in any implementation requires attention and can be estimated through simulation studies not developed in this paper.

## Discussion

This research suggests that the PSM used for computing geographic access of health data, aggregated by ZIP Codes, has several advantages over traditional methods of computing geographic access. The geocoding approaches presented in this paper have their respective benefits and limitations, as follows.

### ZCTA centroid

ZCTA centroids are easy to calculate from ZCTA boundary files, which are publicly available. However, they represent the center of land area, not the center of population, and are based on ZCTA boundaries, which have been called into question for fundamental flaws in the way they are created (by aggregating census blocks based on the majority ZIP Code in each block). If a woman's ZIP Code is in the minority in her block, her block will be assigned to a ZCTA whose code differs from her ZIP Code. In addition, the use of ZCTA centroids as geocodes is deterministic, forcing a single solution, and therefore not representing a set of possible distances or service center destinations for each woman. In cases where some service centers cannot be geocoded to address locations, it is likely that service centers will also be assigned to a point similar to the ZCTA centroid - this can lead to biased distance calculations (i.e., 0 miles) in cases where both the residential and service center location are assigned to the same point; this bias is known as the B-type error in distance estimating methods, which systematically underestimate true road distances [2,19].

### ZIP Code population center

ZIP Code population center points, defined here as the most populated of all populated places in a ZCTA, are more complicated to calculate than ZCTA centroids, but still relatively straight forward. In addition, they improve upon ZCTA centroids because they do not rely on ZCTA boundaries in calculating a center point, instead opting to use populated place population data to increase the likelihood of representing the center of a ZIP Code's population. However, their use is also deterministic, like ZCTA centroids, and could produce biased results if used as a geocode for both residential and service center locations.

### Probabilistic sampling approach

The PSM is more computationally complex than those offered by ZCTA centroid and ZIP Code population center approaches. However, the method capitalizes on the known distribution of the population within the residence region of the subject and computes a distribution of distance estimates for each subject, from which it is possible to calculate the confidence limits of each subject's distance estimate. In addition, this method allows for travel to alternative facilities that are close to the individual, thus offering more insight regarding service access for those with responsibilities for interpreting and using the information. Distance estimates from the PSM method together improve upon the ZCTA centroid, as they represent the center of population within a ZIP Code instead of land area, and improve upon ZIP Code population center points by providing a range of possible locations instead of a single point. Further, this method can be used to weight the sampled blocks based on available demographic information, making it possible to represent the center of different types of populations, such as women of a certain age. The PSM capitalizes on the increased spatial detail that is often available in spatial demographic data compared with the spatial detail used to represent the location of participants whose health is of concern. Finally, this method will likely not result in biased results of 0 distance, as residential and service locations are not assigned to share a single location in space. Selection of the median distance calculated is likely more representative of the true distance traveled, as it is resistant to outlier distances calculated from sampled locations unlikely to be the true residential location of the woman.

Our claim that the PSM is more accurate than other methods used to date, including the two methods used in this paper, rests not on any empirical finding, but on the logic that we have used the spatial distribution of socio-demographic characteristics within the larger residential area and that we have generated the possible distribution of distances to the nearest facility for the person in question. This logic permits us to conclude that we can infer the proportions of cases in which the distance in question will reach any known or given standards of geographical access. Other, non probabilistic methods of distance estimation cannot address this important question. Empirical tests of this method would be valuable in that they would reinforce this assertion and illustrate the scale of the added benefit of the knowledge of the distribution of distances afforded by the use of the PSM. Empirical tests would also contribute to understanding the differential benefit afforded in different geographic settings, such as in urban versus rural settings.

Our limited testing of the PSM algorithm belies the many situations in which it could be used. This type of probabilistic method, which does not require individual addresses, will be increasingly important with increased efforts to protect the confidentiality of health data. Spatial aggregation of the health data of individuals is the most commonly used method to protect the privacy of individual health data. Implemented either at the stage of data acquisition; e.g. "what is your ZIP Code?" or at the stage of data dissemination "location of subject: ZIP Code," the names of the regions perform the function of a geographic mask, preserving the confidentiality of the information promised to the subject and enforced by law [20].

There are a number of situations in which we would anticipate the results of a probabilistic sampling approach to differ even more significantly from ZCTA centroid or ZIP Code center calculations. In Iowa, ZCTAs are of relatively constant geographical size; this is not true throughout the US. In states in which ZIPs/ZCTAs are quite large geographically, one would expect more disparity between calculations using different methods. Also, in cases in which a ZCTA contains more than one highly populated place, calculations using the ZIP Code population center point are likely to differ significantly from calculations using our methodology, especially if these two populated places are far from each other. Finally, in communities that are highly segregated by age, sex, race, ethnicity, or any other characteristic of a respondent used to weight the sampled census blocks, or are zoned to severely separate residential from commercial areas, it is more likely that the PSM will achieve different results than either the ZCTA centroid or ZIP Code center methods.

Table 2 illustrates the many situations in which the PSM algorithm could be used. Typical situations are found where spatial data is collected and disseminated in a spatial hierarchy where smaller areas nest within larger regions which, in turn, nest within even larger regions. A large literature now exists on methods to spatially disaggregate data using symptomatic attributes and geographic information science [21-23]. The last line in this table references one such method which has been widely disseminated recently [21].

**Table 2:** U.S. examples for using the Probabilistic Sampling Method (PSM) for distance estimation

Region of known residence (p)	Smaller areas within the region for which demographic data is available (q)	Typical ratio (q/p)
county	Zip Code, census tract, township, block group, census block	Can vary widely from 36:1 for rural counties to 1000:1 for metropolitan counties.

Zip Code	Census tract, census block group, census block	From 10:1 to 10,000:1

Census tract	Census block group, census block	Variable; 50:1

Census tract	94 Meter grid population data for the U.S. (LandScan U.S.A.)	Highly variable: 1000:1

An important limitation of this study is that the IPV resource facilities to which distances were calculated may not reflect the actual choices a woman would make in seeking services. Factors not taken into account include the type of services available at each facility, whether or not the facility's location is protected for privacy reasons, and the notion that a woman may not want to seek services close to her home, as she may fear for her safety or want to minimize the chances that her seeking of services is made known to people with whom she is acquainted. A more realistic estimation of access to services could be obtained by evaluating the survey responses for each woman in order to determine which services she would likely seek and then interrogating the nature of each facility and using this information to restrict the facilities considered in the analysis to those appropriate to a woman's stated criteria.

Other limitations are also relevant. This study uses Census block population data from the year 2000 while the survey results were collected from 2006-2008. We could have attempted to account for this by utilizing block populations that were several years younger than the woman's age at the time of her survey completion. However, this approach would have assumed a lack of migration (unlikely given the ages of many respondents, who may be moving for school or work). In addition, demographic characteristics beyond age and gender, such as race and ethnicity were not taken into account in the weighting process and could potentially be used in refining the sampling procedure.

The geocoding of the IPV service locations in this study is imperfect, as nearly half of the facilities only provided P.O. Box and ZIP Code - not street address information - and thus their actual locations may not coincide with their geocoded locations. As mentioned previously, they are geocoded to the point representing the largest populated place in the ZIP Code [6]. We chose these points instead of ZCTA centroids to represent locations of the IPV services because we believe they more accurately represent the center of population in the ZIP Code. However, it should be kept in mind that assuming that a domestic violence shelter is located in the center of population is likely a flaw in logic, as they are often intended to have a confidential location, which may be on the outskirts of an urban area. However, without knowing in which direction to offset the location from the urban center, a central location is the most logical solution and is likely superior to using a ZCTA centroid, which is a center of land area and not population.

Following this, a comparison of ZCTA centroid and ZIP Code center distances (Table 1) must include an awareness of the effect of the service center geocode. In cases in which the service center is geocoded to the ZIP Code population center point *and *falls in the same ZCTA as the woman's residence, ZCTA centroid calculations are in effect a calculation of the distance between the ZCTA centroid and ZIP Code center; distance calculations using ZIP Code centers would be zero. In a larger sense, our geocoding of service centers - while necessary due to limited available information - results in distance estimation error, especially given the high proportion of distances that are quite small. This is not a problem that is likely to be rectified, given the necessity of maintaining the confidentiality and security of certain IPV shelters.

Also in regard to the ZIP Code as geocode, we assume that the ZIP Code reported by a woman will correspond geographically to the ZCTA represented by the same 5-digit code. However, this may be a limitation as ZIP Codes and ZCTAs are subject to spatial misalignment. ZCTAs are constructed from all US census blocks where the majority of residents have a particular ZIP Code. If a survey respondent happens to report a ZIP Code that is in the minority in her block, she may be assigned to a ZCTA that does not actually contain her address. As no indication of the accuracy of ZIP Code to ZCTA assignment is publicly available, we cannot address this limitation in our methodology.

Finally, while implementing this method, we discovered three cases in which ZCTA population data contained no women of the respondent's age; in these cases, it was impossible to perform the procedure, and thus we deleted these three records. These scenarios, while few, could be due to the temporal misalignment between the US Census population data (2000) and our survey data (2006-2008) or they could be due to the practice of the U.S. Census to perturb census attribute information between census blocks to prevent users of census data from imputing the attributes of individuals when small numbers of people are found in the cells of the data tables they publish.

## Conclusions

Our findings indicate that the approach used to estimate the geographic location of both services and potential service seekers can affect the estimation of distances between services and service seekers, especially at shorter distances representing more urban areas, and can therefore affect determinations of geographic accessibility of services. Examples of studies that have used centroid to centroid of ZIP Codes to estimate distances to health facilities include Athas et al. (2000) and Onega et al. (2008) to estimate travel distances to radiation therapy and other cancer treatment locations [24,25]. While data sources and methodological approaches are imperfect, it is possible to improve upon this traditional approach of using the ZCTA centroid as the geographic code for a service center and for a service seeker. Results of previous research are consistent with this conclusion.

The idea of using demographic information available for smaller areas than the larger area for which a case location is known has been used in previous research. Hewko et al. (2002) computed the weighted average postal code distances to nearest facilities by disaggregating demographic characteristics of neighborhoods by the smaller census postal codes in Canada that contained them [26]. More recently Apparicio et al. (2008) made a comprehensive comparison of different methods of disaggregating locations to finer census geography [27], while Berke and Shi compared four methods of assigning population within Zip Codes for estimating distance to nearest cancer centers in Arizona and New Hampshire [4]. Henry and Boscoe (2008) cite eight studies that used the demographic characteristics of finer level census geographies to assign geographic locations to cases for which only their larger geographic areas were known [28]. Shi (2007) used the population data in the 800 meter square grid of the LandScan™ database in rural areas of New Hampshire to constrain the random spatial allocation of address data within Zip Code areas for which only Post Office Box numbers were known [29]. His purpose was to evaluate the effect of such spatial imputations on the characteristics of probability maps in kernel density maps of lung cancer incidence. Luo et al. (2010) used Monte Carlo simulation methods similar to those used by Henry and Boscoe (2008) and Shi (2007) to generate a large number of possible distributions of breast cancer cases from the Zip Code to the census block level [30].

In this paper we have taken this approach one step further by using the Monte Carlo simulation of possible locations of the disaggregated spatial locations to compute confidence limits for each imputed distance to a given facility. Unlike previous studies where the purpose of simulating locations of cases was to determine the effect of such estimates on the characteristics of disease maps such as the identification of disease clusters or on general spatial accessibility measures, our purpose is to estimate the likely distribution of possible distances on particular cases where the issue is the spatial behavior of individuals. For this purpose it is necessary to know the range of distances within which the true distance may lie. It is often known that people are willing to travel a certain distance to use a given service. Using the distance confidence limits, it will be possible to estimate the number of people in a given group who will be within the given distance of any service in question.

The methodology used for this project is applicable to any study that seeks to calculate a distance from a person's residence to a service location when the only address information available for the person is the ZIP Code. Thus, this methodology could be widely used in improving the estimation of geographical accessibility. Our comparison of distances calculated when using ZCTA centroids, ZIP Code population centers, and our PSM approach illustrates the variation in estimated distances depending on the geocoding approach chosen. This variation has implications for determinations of geographic "access" to services and for service provision.

## Competing interests

The authors declare that they have no competing interests.

## Authors' contributions

KMMB undertook data analysis and contributed to interpretation of results. AFS and CP-A oversaw data collection. ABW contributed to analysis and interpretation of results. GR conceived of the methodology and contributed to interpretation of results. All authors read and approved the final manuscript.
